# Machine-learning, MRI bone shape and important clinical outcomes in osteoarthritis: data from the Osteoarthritis Initiative

**DOI:** 10.1136/annrheumdis-2020-217160

**Published:** 2020-11-13

**Authors:** Michael A. Bowes, Katherine Kacena, Oras A. Alabas, Alan D. Brett, Bright Dube, Neil Bodick, Philip G Conaghan

**Affiliations:** 1 Imorphics Ltd, Manchester, UK; 2 BioBridges, Wellesley, Massachusetts, USA; 3 Leeds Institute of Rheumatic and Musculoskeletal Medicine, University of Leeds, and NIHR Leeds Biomedical Research Centre, University of Leeds School of Medicine, Leeds, UK; 4 Clinical Research and Medical Affairs, Flexion Therapeutics. Inc, Burlington, Massachusetts, USA

**Keywords:** osteoarthritis, magnetic resonance imaging, knee osteoarthritis

## Abstract

**Objectives:**

Osteoarthritis (OA) structural status is imperfectly classified using radiographic assessment. Statistical shape modelling (SSM), a form of machine-learning, provides precise quantification of a characteristic 3D OA bone shape. We aimed to determine the benefits of this novel measure of OA status for assessing risks of clinically important outcomes.

**Methods:**

The study used 4796 individuals from the Osteoarthritis Initiative cohort. SSM-derived femur bone shape (B-score) was measured from all 9433 baseline knee MRIs. We examined the relationship between B-score, radiographic Kellgren-Lawrence grade (KLG) and current and future pain and function as well as total knee replacement (TKR) up to 8 years.

**Results:**

B-score repeatability supported 40 discrete grades. KLG and B-score were both associated with risk of current and future pain, functional limitation and TKR; logistic regression curves were similar. However, each KLG included a wide range of B-scores. For example, for KLG3, risk of pain was 34.4 (95% CI 31.7 to 37.0)%, but B-scores within KLG3 knees ranged from 0 to 6; for B-score 0, risk was 17.0 (16.1 to 17.9)% while for B-score 6, it was 52.1 (48.8 to 55.4)%. For TKR, KLG3 risk was 15.3 (13.3 to 17.3)%; while B-score 0 had negligible risk, B-score 6 risk was 35.6 (31.8 to 39.6)%. Age, sex and body mass index had negligible effects on association between B-score and symptoms.

**Conclusions:**

B-score provides reader-independent quantification using a single time-point, providing unambiguous OA status with defined clinical risks across the whole range of disease including pre-radiographic OA. B-score heralds a step-change in OA stratification for interventions and improved personalised assessment, analogous to the T-score in osteoporosis.

**Key messages**

**What is already known about this subject?**

- There is a huge unmet need for accurate and reliable assessment of osteoarthritis (OA) status.
- MRI has demonstrated much more pathology but has been largely constrained to reader-dependent semiquantitative assessment.
- Machine-learning enables accurate, reader-independent quantification and we have previously demonstrated it can measure a characteristic OA three-dimensional bone shape with good precision.

**What does this study add?**

- Through application of machine learning, this study has provided a new highly reliable and precise measure of OA status, a quantified 3D femur bone shape termed the B-score.

**How might this impact on clinical practice or future developments?**

- B-score should enable improved stratification for interventions, accurate classification across the range of OA severity and improved personalised assessment, analogous to the role of the T-score in osteoporosis.

## Introduction

Osteoarthritis (OA) is a serious disease resulting in pain, loss of function and reduced quality of life and represents a major public health problem.^1^ The pathophysiology of OA involves multiple tissues, with deterioration of both cartilage and bone considered integral to the OA process.^2^ End-stage disease can be successfully treated with joint replacement, but there has been limited progress with interventions that address earlier OA stages.

OA structural pathology has conventionally been assessed using X-rays. Radiographic determination of OA structural status is imprecise due to its dependence on acquisition method and reader reliability.^3^ The most common scoring system, the semiquantitative Kellgren-Lawrence grade (KLG, scored 0–4), assesses cartilage and bone as well as (indirectly) meniscal changes.^4^ Semiquantitative radiographic assessment has driven our understanding of structure-symptom relationships,^5^ demonstrating associations at group, but not at individual patient level.

MRI has enabled detailed understanding of three-dimensional OA structural pathology and revealed multiple pathologies not evident on X-rays. MRI provides direct quantitative assessment of cartilage and bone^6 7^ and the most responsive imaging biomarkers. However, there remains a strong need for validated surrogate measures of clinically important outcomes, which provide OA status from a single time point, without longitudinal evaluation.

In areas such as hypertension and diabetes, the provision of a single, quantitative measurement has provided breakthroughs in clinical management and drug discovery. In the management of osteoporosis, the dual-energy absorptiometry-based T-score replaced imprecise and insensitive measures based on radiographic bone assessment and photon absorptiometry, creating a single standard measure.

In the field of clinical imaging, the appearance of a tissue can be learnt and then applied to automatically find and delineate that tissue in new, unseen images.^8^ Importantly, this approach is agnostic, being independent of prior expert opinion. Statistical shape modelling (SSM), a type of supervised machine-learning, employs principal component analysis to reduce complex 3D geometric shapes to a single metric value.^9^ Using SSM, we have identified a characteristic OA 3D bone shape, incorporating osteophyte ridge formation and widening and flattening of the articular surfaces. This bone shape predicts radiographic onset of OA,^10^ is associated with radiographic structural progression^11^ and discriminates knees with OA from non-OA.^12^ In each of these studies, the femur had the greatest discrimination and responsiveness, and we have focused this study on femur shape, here termed ‘B-score’. To determine the value of B-score as a measure of OA status, we examined its precision, relationship with the existing radiographic standard (KLG) and explored the relationships of both B-score and KLG with clinically important outcomes: pain, function and total knee replacement (TKR) surgery.

## Methods

### Quantifying tissue shape

#### Patient image data

Data were obtained from the Osteoarthritis Initiative (OAI), a multicentre, longitudinal, prospective observational study of knee OA; bilateral knee MR images were collected in a standardised way together with clinical data from 4796 individuals with, or at risk of developing knee OA.^13^ Data are publicly available at https://data-archive.nimh.nih.gov/oai/.

High-resolution sagittal 3D dual-echo at steady-state water-excitation (DESS-we) knee MRI images were acquired on recruitment into the OAI and at 1, 2 and 4 year timepoints, using a 3T MRI system (MAGNETOM Trio, Siemens Healthcare, Erlangen, Germany). Image acquisition parameters have been published in detail.^14^

#### Statistical shape modelling

Femur bones were automatically segmented from DESS-we images using active appearance models (AAMs), a type of SSM trained to search images, provided by Imorphics (Manchester, UK). AAMs are proven technology that can segment knee bone surfaces with submillimetre accuracy.^12 15^ AAMs were constructed using a training set, from DESS-we images, selected to provide examples of all stages of OA.^16^

We constructed an ‘OA vector’, defined as the line passing through the mean shape of a population with OA (OA Group, defined as all knees with KLG ≥2 at all four time points of 0, 1, 2 and 4 years) and a population without OA (Non-OA Group, defined as those with KLG of 0 at each of the same time points).

#### B-score

**Figure 1:**
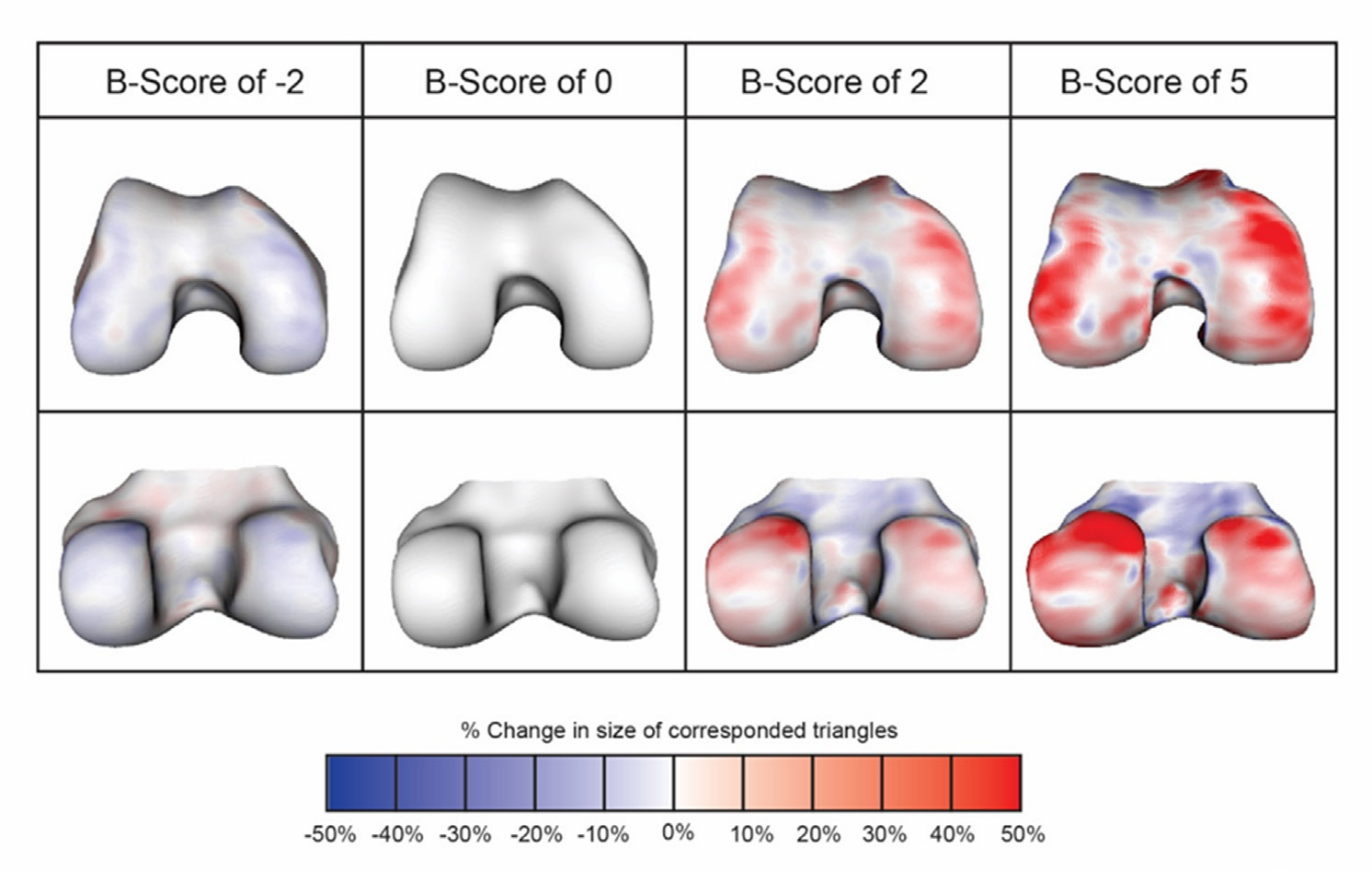
Figure shows change in shape for the anterior femur (top row) and posterior femur (bottom row), for various B-scores. Red indicates where there is an increase in size (locally calculated, based on anatomically corresponded triangles from the shape model), and blue indicates decrease in size (locally); scale shows percentage in area size change of each triangle. Change tends to be greatest around the edge of the cartilage plate (osteophyte region), but it also occurs in central subchondral regions where the bone flattens out.

Distances along the OA vector are termed ‘B-score’, with the origin (B-score 0) defined as the mean shape of the Non-OA Group for each gender. 1 unit is defined as 1 SD of the Non-OA Group along the OA vector (positive values towards the OA Group). Representative examples of differences in femur bone shape at various B-scores, and a heat map of the areas which change most with increasing B-score are shown in figure 1. The range of B-scores in the Non-OA Group was defined as the 95% confidence limits of B-scores in this group, being ±1.96; this enabled delineation of the Non-OA range of B-scores in figures and analysis. Expanded details of the methods for AAM search and construction of B-score are provided in online supplemental methods.

10.1136/annrheumdis-2020-217160.supp1 Supplementary data

### Measurement repeatability

All visually acceptable DESS-we images from the OAI retaken on the same day were assessed; a test-retest set (1 week apart) of those with definite OA were also analysed.^12 16^ Repeatability (smallest detectable difference, SDD) was calculated as the 95% limits of agreement between the two image measurements, using the Bland-Altman method.

KLG reading was performed in the OAI using carefully acquired radiographs, with the knee positioned using a custom-designed frame allowing for a standard knee flexion angle and reporting position of the X-ray source.^17^ Two expert readers independently assessed each radiograph; differences were adjudicated by a group including a more senior reader.

## Statistical analysis

All analyses were conducted using SAS V.9.4 (Cary, North Carolina, USA). Values for the associations with clinical outcomes are presented as proportion of the relevant population; referred to throughout as risk of a clinical outcome.

### Pain by B-score and KL grade

Pain was assessed using the 7-day pain severity numeric rating scale (NRS, 0–10). Current pain was defined as NRS score at baseline, future pain as the median value of all later timepoints (up to 8 years, average follow-up 5 years). Knees were categorised as moderate pain (score ≥4) and severe pain (score ≥8).^18 19^ As a sensitivity analysis, we assessed WOMAC-A pain (0–20 scale, moderate ≥4 and severe ≥8). Logistic regression analyses were performed for current and future pain as defined above against either KLG or baseline B-score, with no additional covariates.

#### Function by B-score and KL grade

Function was assessed using WOMAC function score (0–68), for the knee with the highest B-score per person. Current function was defined at baseline, future function as the median value at all later timepoints (follow-up as for pain). Moderate functional limitation was defined as ≥20 and severe as ≥35.^20 21^ Logistic regression analyses were performed for current and future function as defined above against either KLG or baseline B-score, with no additional covariates.

#### Total knee replacement by B-score and KL grade

KL grade and B-score were independently assessed to determine predictors of TKR at any point during the follow-up period for an individual knee, defined as having an adjudicated TKR within a follow-up period of up to 8 years. This was assessed by modelling TKR as outcome against B-score and KLG separately using logistic regression models.

#### Logistic regression of KLG by B-score quartiles

To assess whether B-score provided additional information over KLG, two modelling approaches were considered. In the first, individual KLG groups were subdivided into quartiles based on B-score and assessed for the five clinical outcomes of current and future pain and function, and TKR, using logistic regression. The second approach involved initially modelling each outcome as described previously with KLG, then adding B-score to each model and assessing whether the regression coefficient for B-score was statistically significant and then calculating the resulting area under the curve (AUCs) for the combined models.

#### Confounders of B-score and risks of clinical outcomes

Potential confounders of the relationship between B-score and the risks of current pain, function and TKR were investigated by adjusting the models for age, sex, ethnicity, body mass index (BMI), alignment, previous knee surgery, non-steroidal anti-inflammatory drugs (NSAIDs) use and smoking status. A description of these variables is shown in the online supplemental methods section.

## Results

### Participant characteristics

Table 1 provides demographic and baseline characteristics. More than 96% of OAI participants had both knees assessed (total knees n=9433). Age ranged from 45 to 79 years. Median BMI was 28 kg/m^2^ (range, 16.9–48.7).

**Table 1:** Demographic and baseline characteristics

Parameter	Males N=1992	Females N=2799	Combined N=4791
Knee MRIs in the OAI dataset at baseline	n=1992	n=2799	n=4791
Both right and left	1929 (97)	2713 (97)	4642 (97)
Right only	37 (2)	49 (2)	86 (2)
Left only	26 (1)	37 (1)	63 (1)
Age (y)	n=1992	n=2799	n=4791
Mean (SD)	60.9 (9.5)	61.3 (9.0)	61.2 (9.2)
Median percentile (25th, 75th)	59 (53 to 70)	61 (54 to 69)	61 (53 to 69)
Min, Max	45 to 79	45 to 79	45 to 79
Race	n=1989	n=2797	n=4786
White	1666 (84)	2122 (76)	3788 (79)
Black or African American	276 (14)	595 (21)	871 (18)
Asian	13 (1)	32 (1)	45 (1)
Other non-white	34 (1)	48 (2)	82 (2)
Current cigarette smoker	n=1964	n=2766	n=4730
No	987 (50)	1513 (55)	2500 (53)
Yes	977 (50)	1253 (45)	2230 (47)
Use of NSAIDs at Baseline	n=1983	n=2796	n=4779
Yes	463 (23)	720 (26)	1183 (25)
No	1520 (77)	2076 (74)	3596 (75)
BMI (m/kg^2^)	n=1990	n=2797	n=4787
Mean (SD)	28.8 (4.15)	28.5 (5.27)	28.6 (4.84)
Median percentile (25th, 75th)	28.5 (25.7 to 31.5)	28.1 (24.4 to 32.0)	28.2 (25.1 to 31.7)
Min, Max	18.3 to 44.6	16.9 to 48.7	16.9 to 48.7

All values are n (%) unless stated.

*BMI denotes body mass index, MRI magnetic resonance imaging, NSAIDS nonsteroidal anti-inflammatory drugs, and OAI Osteoarthritis Initiative.

### Repeatability

A total of 139 knees were imaged twice on the same day within the OAI: the repeatability (SDD) of B-score in this group was 0.251 (B-score units). This group was representative of the whole OAI dataset (86 female, KLG 0, 1, 2, 3, 4 as fraction: 33%, 20%, 31%, 12%, 4%, BMI mean (SD) 30.3 (5.23); mean age (SD) 62.7 (9.45). A total of 35 knees were imaged in the test-retest set, at baseline and 1 week: SDD of B-score in these images was 0.254. This represents 2.5% of the likely range of B-scores (−3 to +7 in this study).

### Relationship of B-score with KL grade

**Figure 2:**
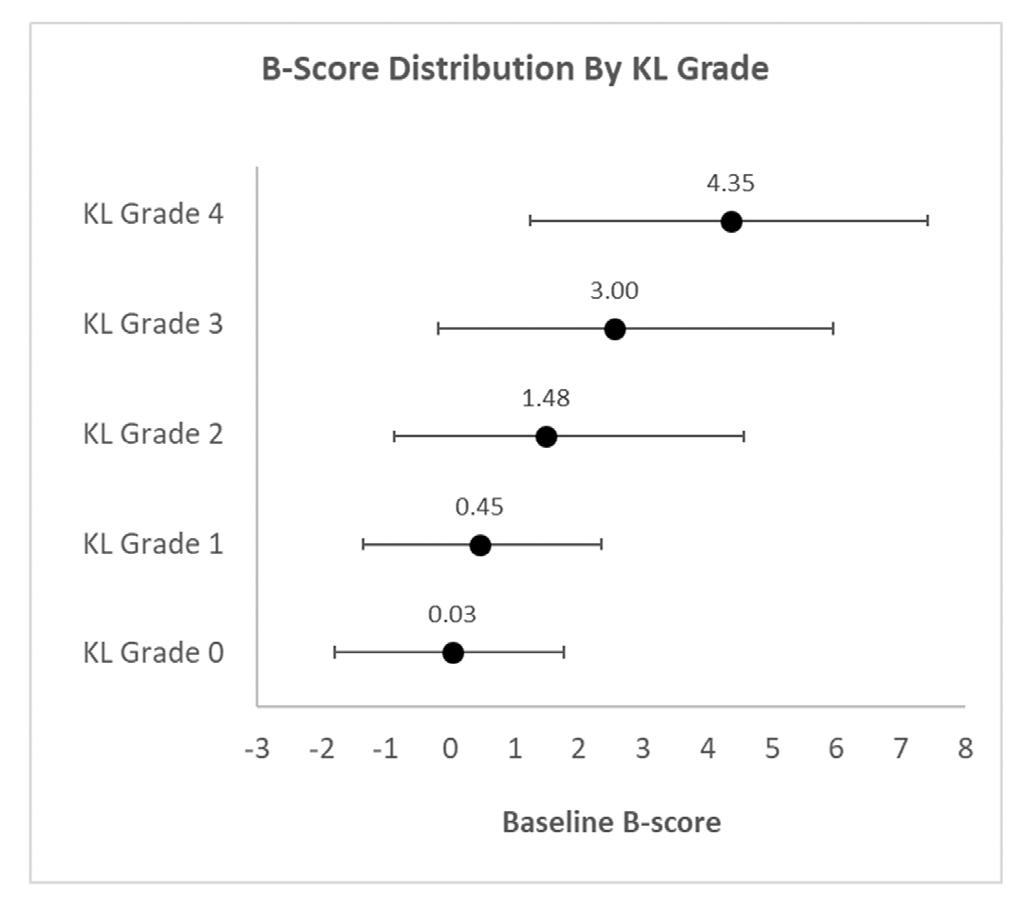
Distribution of B scores by KL grade are displayed for males and females (mean and 95% CIs for each grade). Mean B score for each KL grade is noted above each line.

Distribution of B-score by KLG is shown in figure 2. There was a large range of B scores for each KLG, reflecting the increased measurement sensitivity of the measure, with B-score range increasing with KLG. Mean B-score had a non-linear association with KLG, increasing more rapidly at grades 3 and 4; CIs were wider with increased KLG. For example, the 95% confidence limits of B-score for a KLG3 knee (n=1237) were –0.2 and +6.0. 3.4% of KLG0 knees had B-scores greater than the non-OA range, KLG1:7.9%, KLG2:33.1% KLG3:57.6%, KLG4:89.3%. Proportions of B-score bins classified by KLG are shown in online supplemental table S4 and online supplemental figure S3.

### KLG and clinically important outcomes

**Figure 3:**
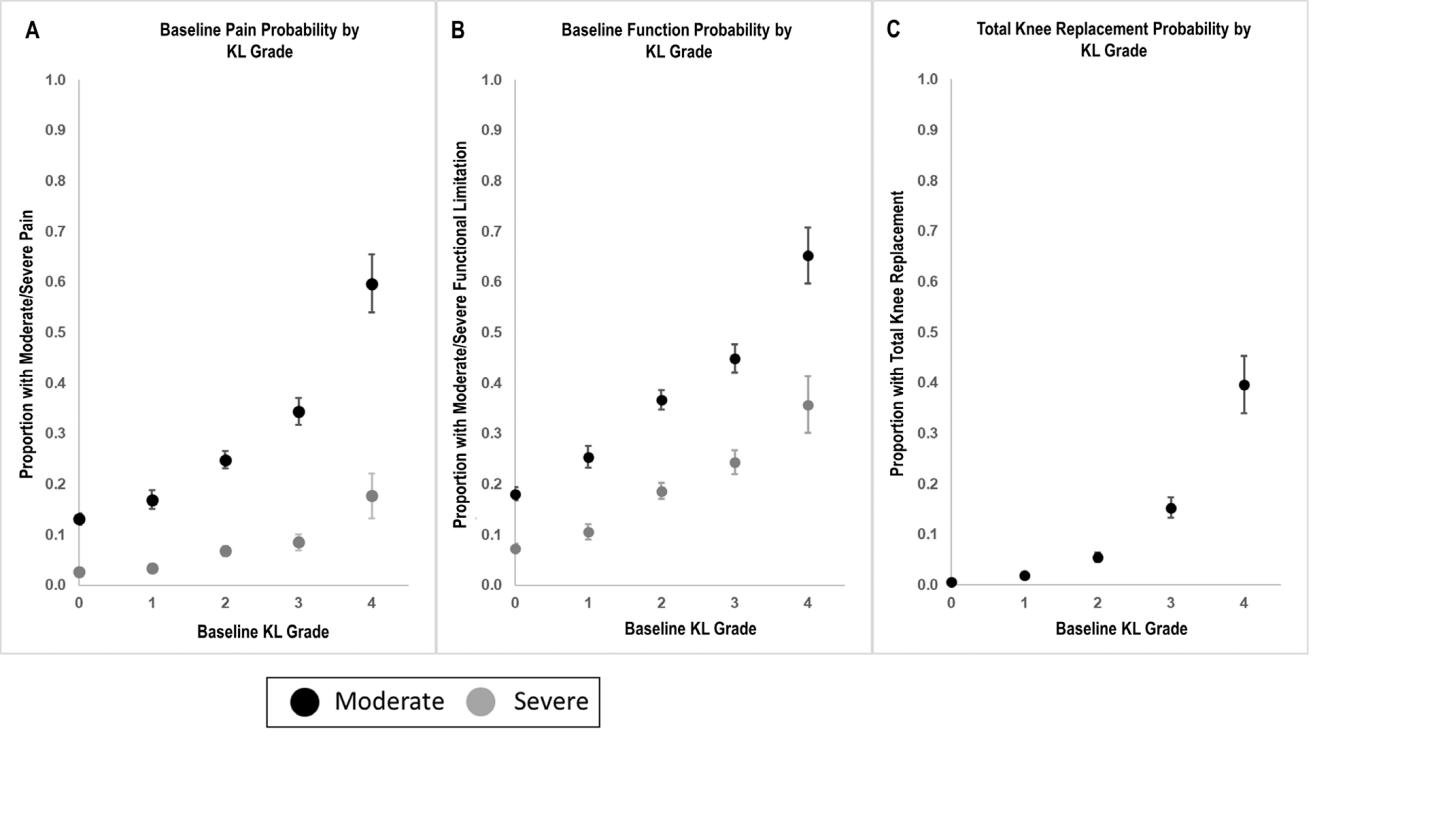
Error bars show 95% confidence limits for each measure. Pain: moderate or greater pain was defined as NRS pain ≥4 on the 10-unit scale (black points); severe pain as NRS pain ≥8 (grey points). Function: moderate or greater limitation of function was defined as function ≥20 on the 68-point WOMAC function scale (black points); severe loss of function was defined as ≥36 (grey points). TKR—risk of total knee replacement over follow-up period (up to 8 years, average follow up 5 years).

The risk of moderate knee pain or limitation of function increased across the range of KLG from around 10% to around 60%; this was not linear, and risk increased more rapidly between KLG 3 and 4 (figure 3). Risks of severe knee pain or severe limitation of function also increased from 2% to 15% and 8% to 35%, respectively. Risk of TKR increased in a curvilinear manner, with risk increasing approximately 2.5-fold for each increase in KLG. Risk of future pain and function are shown in online supplemental figure S1.

### B-score and clinically important outcomes

**Figure 4:**
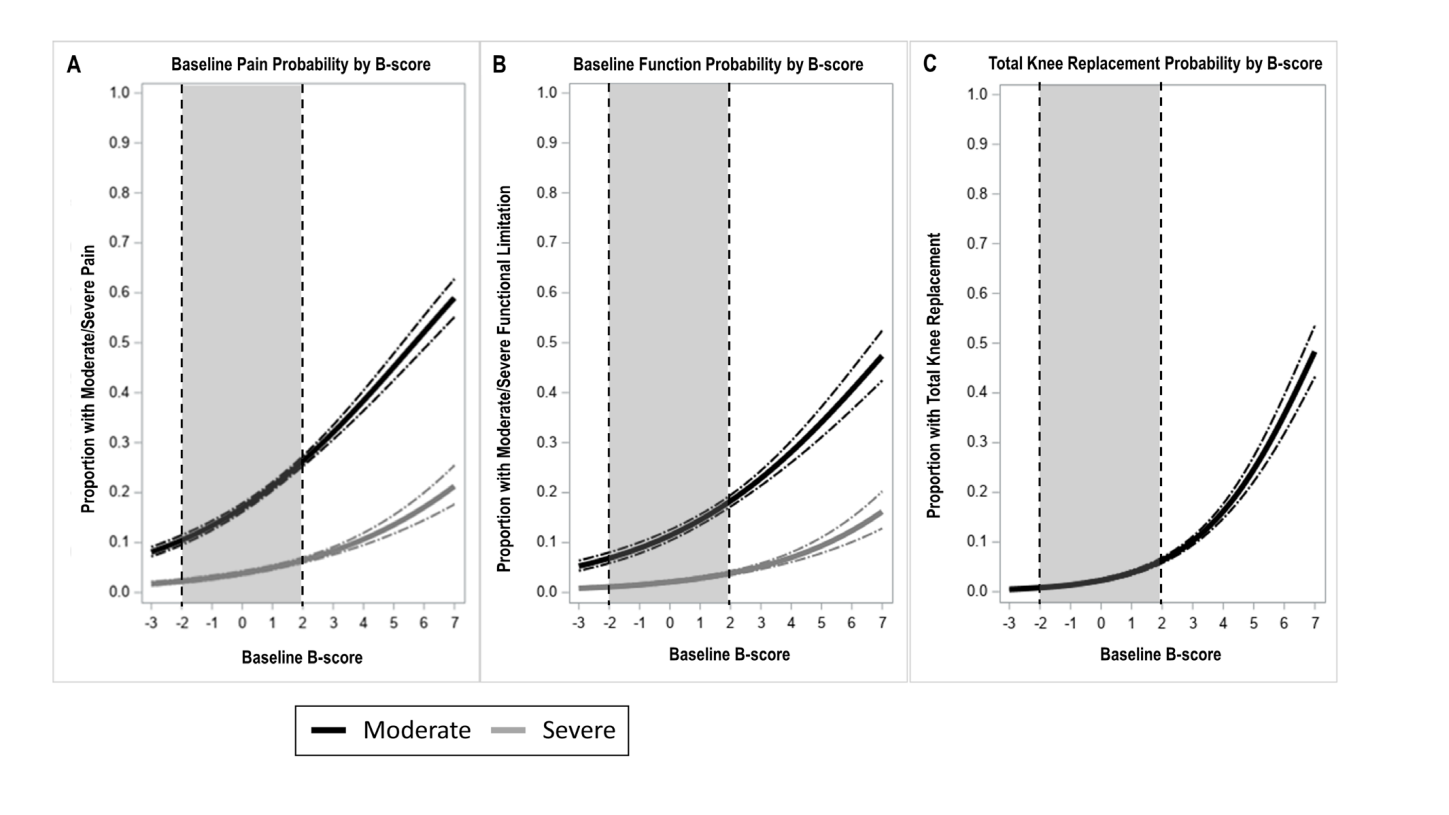
Error bars show 95% CIs for each measure. Moderate or greater pain was defined as NRS pain ≥4 on the 10-unit scale (black lines); severe pain as NRS pain ≥8 (grey lines). Moderate or greater limitation of function was defined as function ≥20 on the 68-point WOMAC function scale (black lines); severe limitation of function was defined as ≥36 (grey lines). TKR—risk of total knee replacement over follow-up period (up to 8 years, average follow-up 5 years). Limits of non-OA group B-scores are provided using a dotted line and greyed area.

The risks of moderate knee pain or loss of function increased across the range of B-score from around 10% to around 60% and are curvilinear (figure 4 and online supplemental table S1). Risks of severe knee pain or severe function limitation increased similarly. Risk of TKR also increased similarly. Risks of future pain and function are shown in online supplemental figure S1. The distribution of pain, function and other OA-related factors at baseline is shown in online supplemental table S2. AUCs for the relationship of B-score and all five outcomes were comparable with those found for KLG and those outcomes (online supplemental table S3).

### Additional information provided by B-score

Within KLG2-4, ORs for all clinical outcomes varied significantly between lowest and highest B-score quartiles (p<0.001) (for KLG3 knees, see table 2). No statistically significant differences were found between lowest and highest quartiles in KLG 0 and 1 knees. In terms of discrimination, addition of B-score resulted in improvement in the AUCs in all models, although of small magnitudes (online supplemental table S3), while the regression coefficient for B-score was statistically significant (p<0.05) in all models.

**Table 2:** ORs and 95% CIs for B score quartiles among KLG 3 & 4 knees, compared with the lowest B score quartile, for all current and future clinical outcomes

Outcome	B- Score Quartile 2	B-ScoreQuartile 3	B-scoreQuartile 4
Pain moderate - current	1.36 (0.95,1.94)	1.76 (1.24,2.49)***	2.4 (1.69,3.4)***
Pain severe - current	1.43 (0.67,3.05)	3.13 (1.59,6.16)**	3.54 (1.8,6.93)***
Function loss moderate - current	1.67 (1.12,2.51)*	1.91 (1.28,2.86)**	2.35 (1.58,3.49)***
Function loss severe - current	1.22 (0.5,2.99)	2.66 (1.21,5.84)*	2.03 (0.89,4.63)
Pain moderate - future	1.95 (1.33,2.86)***	2.54 (1.74,3.69)***	3.18 (2.18,4.62)***
Pain severe - future	1.25 (0.49,3.21)	3.28 (1.46,7.4)**	3.62 (1.61,8.14)**
Function loss moderate - future	1.61 (0.99,2.62)	2.83 (1.79,4.48)***	3.52 (2.23,5.55)***
Function loss severe - future	1.23 (0.37,4.06)	2.95 (1.05,8.29)*	2.16 (0.73,6.41)
Total knee replacement	1.21 (0.73,2.01)	1.51 (0.93,2.47)	2.58 (1.62,4.09)***

*P<0.05, **p<0.01, ***p<0.001.

### Increased discrimination of all risks, using B-score at individual patient level

The increased utility of B-score is demonstrated by considering a KLG3 knee. The mean(CI) risk of a moderately painful knee based on this KLG was 34.4 (31.7 to 37.0)%. B-score within KLG3 knees ranged (95% CI) from 0 to 6; if the knee had a B-score of 0 the risk of a moderately painful knee was 17.0 (16.1 to 17.9)% while for a B-score of 6 it was 52.1 (48.8 to 55.4)%. The risk of a moderate limitation of function for a KLG3 was 20.6 (18.2 to 22.9)% if the knee had a B-score of 0 the risk of moderate function limitation was 11.4 (10.4 to 12.5)% while for a B-score of 6 it was 40.6 (36.6 to 44.6)%. For TKR, KLG3 knee had risks of 15.3 (13.3 to 17.3)%, whereas B-score 0 had negligible risk of TKR 2.3 (2.0 to 2.6)% and B-score six had a risk of 35.6 (31.8 to 39.6)%.

### Confounders of, and additional information provided by, B-score

After adjustment for covariates the effect sizes from regression were still classified as ‘small’ for the risk of pain, function or TKR (online supplemental table S1).

## Discussion

Machine-learning has made possible the development of a quantitative measure of OA status; we have termed this the B-score. In this large observational cohort, B-score produced logistic regression models for clinically important outcomes, which were very similar in terms of predictive validity to those of the existing radiographic standard, providing construct validity for this new measure. However, by providing a scalar measure enabling at least 40 measurable subdivisions for OA structural change, B-score provides increased discrimination of risk over KLG for all clinically important outcomes. As a fully automated (reader-independent) measurement, B-score allows for rapid analysis of large datasets; and in both clinical trials and routine practice, provides a consistent measurement metric. As a scalar measure (compared with the categorical KLG), B-score permits the use of more powerful statistical methods for analysis.

The primary utility afforded by the precision of B-score is demonstrated by comparison with KLG. We have presented an example for KLG3 in the Results section, demonstrating the benefits conferred by having a range of B-scores within a single KLG. This applies for all KLG, even for a KLG0 knee, (often considered to be normal), for which the mean risk of moderate pain was 12%, while B-score risk range (−2 to +2) was 10%–27%. In day-to-day clinical use, it is unlikely that KLGs can be as consistent and repeatable as those in the OAI, where images are carefully acquired and read. Several studies estimated inter-reader agreement of KLG and found a ‘moderate’ intraclass coefficient of around 0.5–0.7.^22–24^ In practice, this means that a KLG3 knee has an equal chance of being scored as KLG2, 3 or 4. This misclassification profoundly affects the risks exemplified above: a KLG3 knee had a risk of between 13.3% and 17.3% of TKR within 8 years. If the knee is equally likely to be scored as KLG2 or KLG4, then this becomes 4.5%–45.5%, a 10-fold increase in CI.

B-score provides a measure of OA status across the whole range of OA structural severity, including early disease. This is often conceptualised as KLG2, but the findings of the current study show that 31% of those categorised as KLG2 had a B-score within the non-OA range, and KLG0-1 knees included 8% with B-scores above the non-OA range. There is currently no consensus on a definition of ‘early’ OA, and B-score can provide a valuable measure. We have used the 95% CI of those who almost certainly do not have radiographic OA (B-score of ≤ 1.96), and this seems a well-validated basis for a cut-off point. In clinical trials, B-score would provide a reliable stratification tool and has already shown to be a sensitive outcome measure.^25^ A number of therapies, including platelet-rich plasma and hyaluronic acid, are used in early OA,^26^ and their effect on OA structural progression can now be meaningfully assessed. Implications for clinical practice require further consideration, and at present may improve assessment of prognosis more than selection of therapy (given our limited non-surgical therapeutic options). However, B-score may initially provide clinical usefulness in situations where MRI is already commonly performed (eg, sporting injuries or ‘possible early OA’).

It was not the intention of this study to suggest that bone shape pathology is causally related with the clinically important outcomes; bone shape is likely reflecting a broader OA construct. It is widely believed that the clinically important outcomes used in this study are related to age, sex, ethnicity, BMI and alignment, and these covariates are often used as inclusion criteria in OA clinical trials. In this study, these covariates had negligible effects on the ORs of the relationship between B-score, a measure of bone pathology, and clinically important outcomes.

We did perform a number of sensitivity analyses on the choice of symptom cut-points, in the absence of widespread consensus on what constitutes moderate and severe symptoms. As well as using a second tool, (WOMAC pain, see online supplemental figure S2) which showed a similar symptom-structure relationship to the NRS score used in the main paper, we also performed sensitivity testing using values of 7, 8 or 9 as cut-off for ‘severe’ pain, and 32, 34 or 36 as cut-off points for function loss and found that the choice of any of these cut-off points was not an important effect (data not shown).

The strength of this work includes very large patient numbers, but there are limitations. We have not attempted to explore longitudinal change or relationship to cartilage as we focused on the benefits of this new measure at a single time point, and its clear relationships with clinically important outcomes. Our non-OA group, used to set the scale of B-score, was drawn from the OAI with a population aged 45–80, in contrast to the osteoporosis T-score which uses a reference population of healthy young adults. Although we used the DESS-we MR images in this study and have previously demonstrated that the method is applicable to similar MRI sequences,^27^ the method would need validation for other MRI sequences. We used a regression analysis for the risk of TKR, rather than hazard or incident rate analysis, as TKR was a ‘rare’ outcome in our data set, and also to allow the reader to compare estimates in our study (figures 3 and 4). The machine-learning technology can almost certainly be applied to cheaper imaging methods such as CT. Although the method for B-score determination used in this study is proprietary, several methods for bone shape measurement have been published, and the measurement of bone shape is actively being pursued by multiple groups. The bone shape vector revealed here may not hold for very late stages of the disease, where fewer patient numbers were available in this study. When osteophytes begin to carry load directly, they are likely to remodel and may produce shape changes that are less systematic than those reported here.

In conclusion, machine learning has enabled the development of a new objective, precise single time-point measure, B-score, representing OA status. B-score demonstrated similar relationships to clinically important outcomes as the current radiographic standard, but with the increased precision of B-score (providing approximately 10 times more detail on OA structural status), enabling better risk discrimination for clinically important outcomes. B-score should enable improved stratification for interventions and improved personalised assessment, in the same way that bone mineral density and more specifically, the T-score, has done historically for osteoporosis.
