# Supplementary material for: Machine-learning, MRI bone shape and important clinical outcomes in osteoarthritis: data from the Osteoarthritis Initiative
Source: Ann Rheum Dis. 2020 Nov 13;80(4):502–8. doi: 10.1136/annrheumdis-2020-217160 (PMC7958089; doi:10.1136/annrheumdis-2020-217160)
Supplement: Supplementary data [file annrheumdis-2020-217160supp001.pdf]

Supplementary Material: Machine-learning, MRI bone shape and important clinical outcomes in osteoarthritis: data from the Osteoarthritis Initiative

SUPPLEMENTARY RESULTS

Supplementary Table S1. Effect of correcting for covariates (Age, Sex, Race, BMI, Alignment, Previous Knee Surgery, Use of NSAIDS, Smoking Status) on risks of clinically important outcomes

Outcome Variable	Unadjusted Odds Ratio B-score [95% CI] (p-value)	Adjusted Odds Ratio B-score [95% CI] (p-value)
Current NRS Pain		
Moderate Pain	1.322 [1.288,1.358] (<0.0001)	1.153 [1.084,1.227] (<0.0001)
Severe Pain	1.314 [1.260,1.370] (<0.0001)	1.184 [1.079,1.300] (0.0004)
Current WOMAC Pain		
Moderate Pain	1.322 [1.289,1.357] (<0.0001)	1.204 [1.131,1.281] (<0.0001)
Severe Pain	1.345 [1.300,1.391] (<0.0001)	1.146 [1.060.1.239] (0.0006)
Current Function (worst knee)		
Moderate limitation of function	1.334 [1.300,1.368] (<0.0001)	1.108 [1.033,1.188] (0.0039)
Severe limitation of function	1.333 [1.295,1.373] (<0.0001)	1.257 [1.100,1.436] (0.0008)
Total Knee Replacement	1.694 [1.624,1.767] (<0.0001)	1.653 [1.508,1.811] (<0.0001)

Potential confounders of the relationship between B-score and the risks of current pain, function and TKR were investigated by adjusting the models for age, sex, ethnicity BMI, alignment, previous knee surgery, NSAID use and smoking status. A description of these variables is shown in the Supplementary Methods section below.

Bowes MA, *et al. Ann Rheum Dis* 2021; 80:502–508. doi: 10.1136/annrheumdis-2020-217160

Supplementary Table S2. Osteoarthritis Indicators at Baseline

Parameter	Males n = 3921 knees	Females n = 5512 knees	Combined n = 9433 knees
**WOMAC-A (pain) at baseline, n (%)**	n=3921	n=5512	n=9433
0 to <4: No pain to low pain	2983 (76.1)	3942 (71.5)	6925 (73.4)
4 to <8: Moderate pain	644 (16.4)	956 (17.3)	1600 (17.0)
8 or more: Severe pain	294 (7.5)	614 (11.1)	908 (9.6)
**NRS (pain) at baseline, n (%)**			
0 to <4: No pain to low pain	3146 (80.2)	4188 (76.0)	7334 (77.8)
4 to <8: Moderate pain	600 (15.3)	975 (17.7)	1575 (16.7)
8 or more: Severe pain	175 (4.5)	349 (6.3)	524 (5.55)
**Function limitation, n (%)**	n=3921	n=5512	n=9233
0 to <20: No or low limitation	3480 (88.9)	4547 (82.5)	8027 (85.1)
20 to <36: Moderate limitation	356 (9.1)	735 (13.3)	1091 (11.6)
36 or more: Severe limitation	85 (2.2)	230 (4.2)	315 (3.3)
**Alignment (degrees)**	n=3861	n=5393	n=9254
Mean (SD)	0.81 (2.98)	-1.07 (2.76)	-0.28 (3.00)
Median percentile (25th, 75th)	0.50 (-1, 3)	-1 (-3, 0)	-1 (-2, 1.5)
Min, Max	-11,15	-20, 11	-20, 15
**Previous knee surgery, n (%)**	n=3921	n=5512	n=9433
Yes	693 (17.7)	447 (8.1)	1140 (12.1)
**Kellgren-Lawrence Grade at baseline, n (%)**	n=3705	n=5129	n=8834
0	1496 (40.0)	1927 (37.6)	3423 (38.8)
1	653 (17.6)	924 (18.0)	1577 (17.9)
2	824 (22.2)	1506 (29.4)	2330 (26.4)
3	560 (15.1)	656 (12.8)	1216 (13.8)
4	172 (4.6)	116 (2.3)	288 (3.3)
**B-score at baseline**	n=3921	n=5512	n=9433
Mean (SD)	0.90 (1.77)	1.05 (1.78)	0.99 (1.78)
Median percentile (25th, 75th)	0.61 (–0.23, 1.68)	0.77 (–0.18, 1.93)	0.71 (–0.20, 1.84)
Min, Max	–3.41, 8.69	–3.46, 9.97	–3.46, 9.97

WOMAC denotes Western Ontario and McMaster Universities Osteoarthritis Index.

Bowes MA, *et al. Ann Rheum Dis* 2021; 80:502–508. doi: 10.1136/annrheumdis-2020-217160

Supplementary Table S3. Area under the curve for logistic regression models of B-score and KL grade vs current clinical outcomes.

Outcome	KL grade	B-score and KL grade	B score
Moderate Pain	64.88%	66.41%	63.73%
Severe Pain	65.45%	68.14%	65.28%
Moderate Functional Limitation	66.1%	69.17%	65.0%
Severe Functional Limitation	67.26%	69.94%	67.67%
Total Knee Replacement	82.84%	85.02%	79.5%

Bowes MA, *et al. Ann Rheum Dis* 2021; 80:502–508. doi: 10.1136/annrheumdis-2020-217160

Supplementary Table S4. Proportions of KL grades by B-score, and B-score by KL grade

		KL Grade				
**B-score range**	***n***	**0**	**1**	**2**	**3**	**4**
**< -3 to -2·5**	33	79%	15%	6%	0%	0%
**< -2·5 to -2**	112	77%	17%	4%	2%	0%
**< -2 to -1·5**	221	73%	14%	12%	1%	0%
**< -1·5 to -1**	449	63%	20%	14%	3%	0%
**< -1 to -0·5**	768	60%	22%	15%	3%	0%
**< -0·5 to 0**	1,098	58%	19%	19%	4%	0%
**> 0 to 0·5**	1,258	50%	23%	21%	6%	0%
**> 0·5 to 1**	1,230	44%	23%	25%	7%	1%
**> 1 to 1·5**	994	34%	23%	30%	12%	1%
**> 1·5 to 2**	729	23%	18%	37%	20%	2%
**> 2 to 2·5**	514	14%	13%	45%	26%	2%
**> 2·5 to 3**	371	9%	12%	40%	33%	6%
**> 3 to 3·5**	267	4%	4%	39%	38%	15%
**> 3·5 to 4**	202	0%	0%	50%	42%	8%
**> 4 to 4·5**	169	1%	1%	41%	40%	18%
**> 4·5 to 5**	148	0%	1%	36%	41%	22%
**> 5 to 5·5**	105	0%	0%	26%	47%	27%
**> 5·5 to 6**	71	0%	0%	38%	35%	27%
**> 6 to 6·5**	53	0%	0%	15%	43%	42%
**> 6·5 to 7**	34	0%	0%	15%	56%	29%

Proportions of knees recorded as KL grades 0, 1,2,3,4 for 20 bins of B-score. Note that measurement repeatability supports the use of 40 categories; we have used 20 here to ensure that outer bins contain sufficient numbers. Data are graphically represented in Supplementary Figure S3.

Bowes MA, *et al. Ann Rheum Dis* 2021; 80:502–508. doi: 10.1136/annrheumdis-2020-217160

Supplementary Figure S1. Future (A) NRS pain and (B) functional limitation by B-score

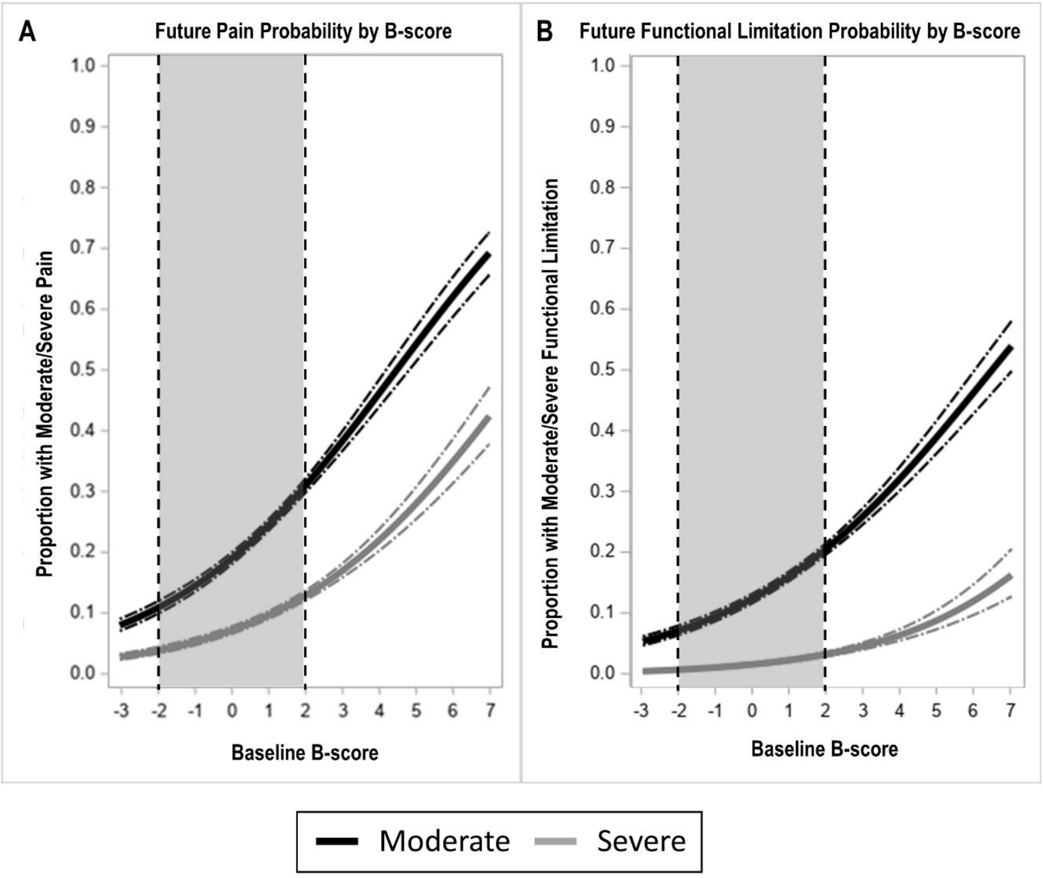

Error bars show 95% confidence intervals for each measure. Moderate or greater pain was defined as NRS pain ≥4 on the 10-unit scale (black lines); severe pain as NRS pain ≥8 (grey lines). Moderate or greater limitation of function was defined as function ≥10 on the 68-point WOMAC function scale (black lines); severe loss of function was defined as ≥20 (grey lines). Limits of Non-OA group B-scores are provided using a dotted line and greyed area. Future values were determined as the median value at all follow-up time points (excluding baseline, up to 8 years, average follow-up 5 years).

Bowes MA, *et al. Ann Rheum Dis* 2021; 80:502–508. doi: 10.1136/annrheumdis-2020-217160

Supplementary Figure S2. Current (A) and future (B) WOMAC pain by B-score

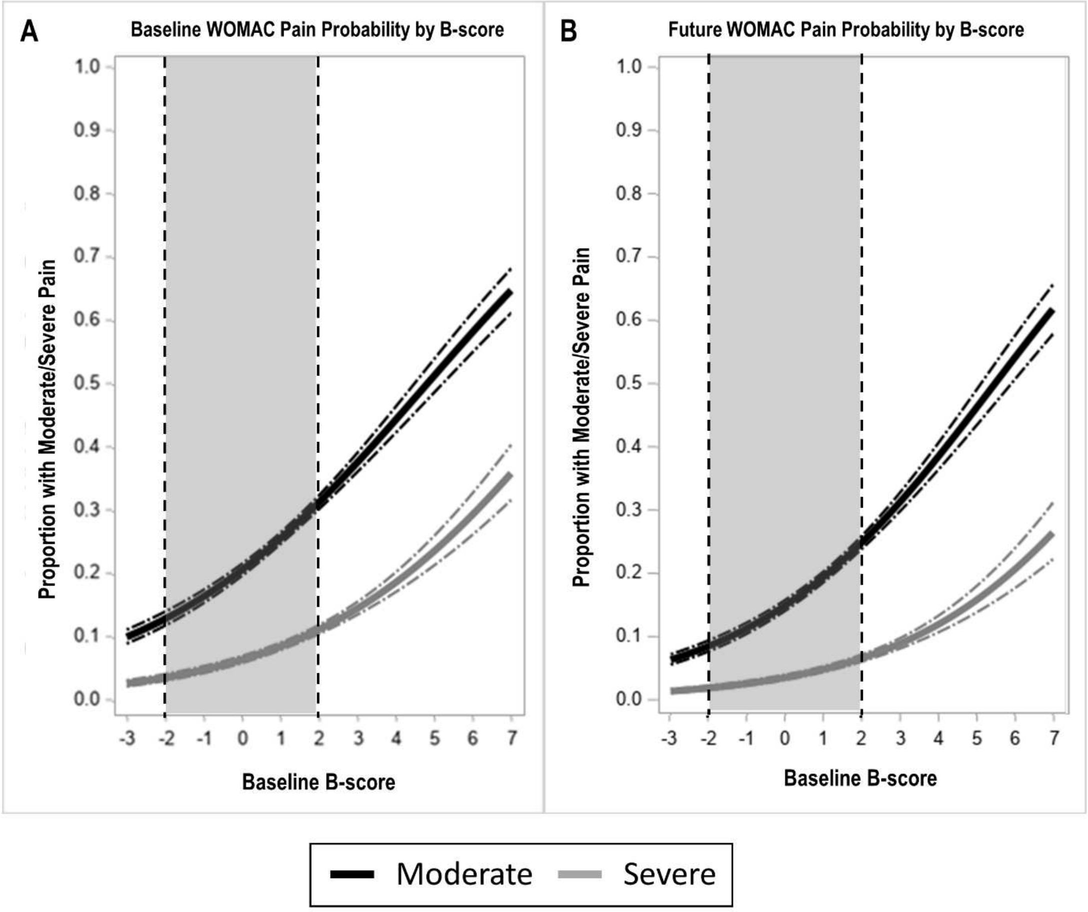

Error bars show 95% confidence intervals for each measure. Moderate or greater pain was defined as WOMAC pain ≥4 on the 10-unit scale (black points); severe pain as WOMAC pain ≥8 (grey points). Limits of Non-OA group B-scores are provided using a dotted line and greyed area.

Supplementary Figure S3. Distribution of B-scores by KL Grade

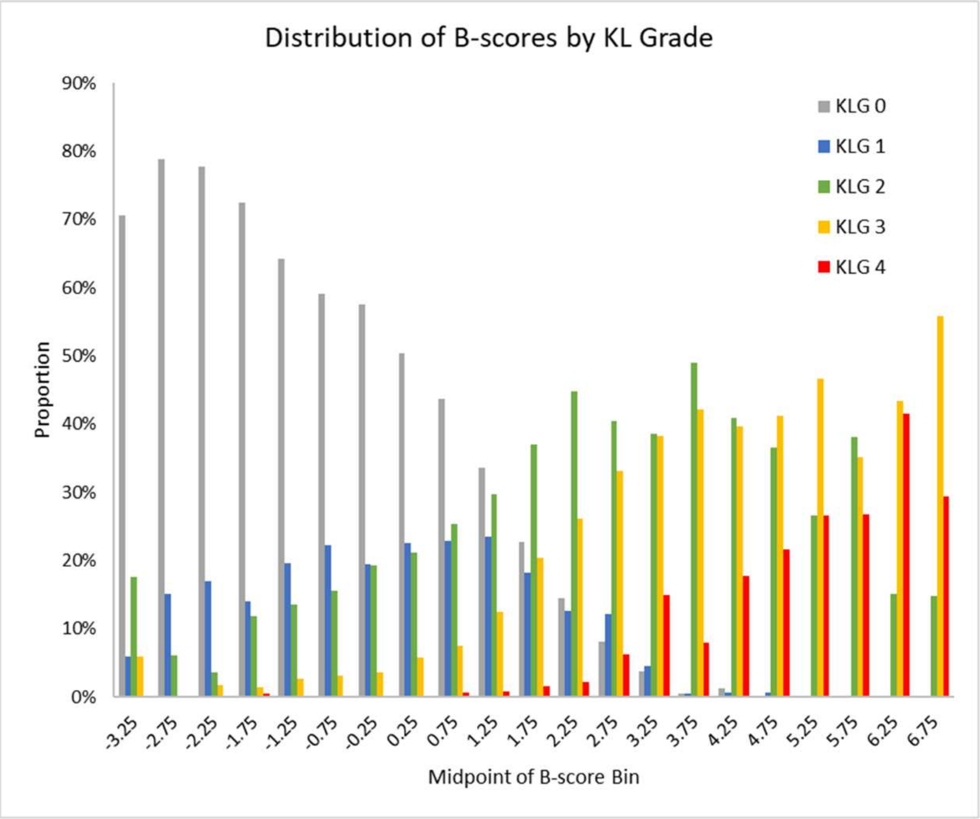

Graphic representation of data in Supplementary Table S4

SUPPLEMENTARY METHODS

Definition of variables and assessment of confounders

All data from the Osteoarthritis Initiative (OAI) that were utilised in this study are publicly available at https://data-archive.nimh.nih.gov/oai.

For the different outcomes assessed, the influence of covariates (both confounders and competing exposures) chosen *a priori* from previously established clinical relationships was evaluated. Given the large sample size, both the statistical significance and the size of the estimates were considered. The covariates considered and adjusted for in the regression models were age, sex, BMI, ethnicity, previous knee surgery, alignment, NSAID use and smoking status described in more detail below.

Covariates were coded as recorded by the OAI. Age was modelled as a continuous variable in years, sex was binary (male or female), BMI as a continuous variable in kg/m^2. Ethnicity was categorised as White or Caucasian, Black or African-American, Asian, Other Non-white. Previous knee surgery was modelled as a binary variable coded as zero if participant had no history of previous surgery and one if they reported any previous knee surgery. In the OAI previous knee surgery was defined as "history of knee surgery (including arthroscopy, ligament repair, and meniscectomy)". Alignment was measured using a goniometer and recorded in degrees which was modelled as a continuous variable in degrees. NSAID use was modelled as a binary variable (yes or no). The definition of NSAID use was any use of prescription or non-prescription NSAIDS (e.g., Ibuprofen, Diclofenac, Aspirin…) for joint pain or arthritis for more than half the days of the month in the past 30 days. Smoking status was modelled as a categorical variable with 3 levels (never, current and former).

The variables considered for the regression models were based on *a priori* relationships between the outcomes. For TKR for example, we considered clinically important risk factors such as age, gender, weight, and pain, which may influence the surgeon`s decision to

operate. We also considered whether health insurance could affect the outcome with participants potentially not offered a TKR for financial reasons; however, on exploration of the data we found that 98% of participants that had a TKR had some form of health insurance while 96% of those not having a TKR had insurance.

Tests for interactions

Interactions, including that for age were considered during an initial analysis, but as the differences between univariable and adjusted models showed that the odds ratios represented small effects after adjustment, a parsimonious model was chosen as the final model, excluding interactions.

Statistical Shape Modelling

Femur bones were automatically segmented from DESS-we images using active appearance models (AAMs), a type of SSM trained to search images, provided by Imorphics (Manchester, UK). AAMs are proven technology, which can segment knee bone surfaces with sub-millimetre accuracy [1, 2] [*references 15, 16 respectively in main paper*]. AAMs were constructed using a training set, consisting of expert manual segmentations of DESS-we images, selected to provide examples of all stages of OA. The training set was selected to contain examples of each stage of OA (43 KLG0 and KLG1, 7 KLG2, 28 KLG3, 18 KLG 4) [3] [*reference 17 in main paper*]. Accuracy of bone segmentation was excellent, with point-to-surface accuracy against careful manual segmentation of ± 0.49mm (95% confidence limits of error), and repeatability of all bone measurements was excellent with typical coefficient of variations of 0.4% to 0.6% [1]. Adding additional training examples to the model beyond the

96 examples, with differing degrees of osteoarthritis, did not increase segmentation accuracy.

The construction of an AAM parameterises femur bone shape using principal component analysis. Each time that a femur bone shape is identified within an image using an AAM, the femur bone shape is returned as a set of principal components.

OA Vector

Using the principal components from the AAM, we calculated the mean shape from two populations:

1. The “Non-OA group”, being the group of all knees with KLG0 radiograph reading at 0,1,2 and 4 years in the OAI (n=885), regardless of sex

2. The “OA group”, being the group of all knees with KLG ≥2 at 0, 1, 2 and 4 years (n = 1,713), regardless of sex.

There is no risk of over-training any subsequent models using 2,597 knees, as the only information taken from these populations of knees was the mean shape of the two groups. An “OA vector” was defined as the line passing through the mean shape of the Non-OA group shape, and the OA group (Supplementary Figure S4).

Supplementary Figure S4. Sammon plot illustrating the shape distributions of 600 femurs used in the training set and the OA vector.

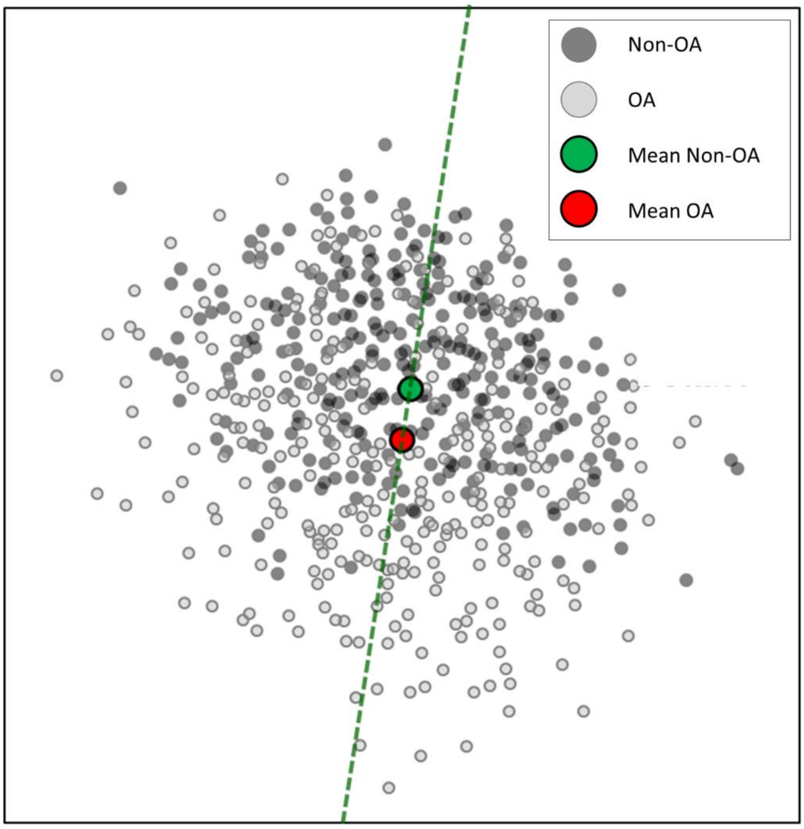

Figure shows the population of the training set, randomly sampled down to 600 points for legibility of figure. A Sammon plot reduces all of the principal component dimensions into 2 dimensions while preserving the distances between shapes as far as possible. Green circle shows the average shape of the Non-OA group (dark grey circles), and red circle the average shape of the OA group (light grey circles). Dotted green line is the OA vector, the line which passes through these two mean shapes. Histograms showing the projection of points from the Non-OA and OA groups onto the OA vector is shown in Supplementary Figure S5 below.

B-Score and sex

Each parameterized femur bone shape was projected orthogonally onto the OA vector to provide a distance along the OA vector. This distance was then normalised as follows: the origin (B-score of 0) was defined as the mean shape of the Non-OA Group for each sex. Means were determined separately for males and females (although the OA vector is constructed using both sexes). Males and females (with or without OA) have systematically different 3D bone shape [4] [*reference not cited in main text*], other than the OA shape

described here, resulting in a systematic difference along the OA vector for each sex. This is corrected, by calculating the means separately for each sex, but continuing to use the OA vector which contains both sexes. The distribution of male and female knees from the Non-OA or OA groups, after the correction are shown in Supplementary Figure S5.

Preparing entirely separate models for sex did not improve classification of OA vs Non-OA, sensitivity to change, and the logistic regression models for pain, function and TKA were indistinguishable from those using a vector containing all males and females (data not shown). As a result, a single vector combining the sexes was used for this study, with the origin corrected separately for males and females. Scale is defined as 1 standard deviation of the distribution of the Non-OA Group along the OA vector (with positive direction being toward the OA Group).

Supplementary Figure S5: Distribution of Non-OA and OA groups following correction of means.

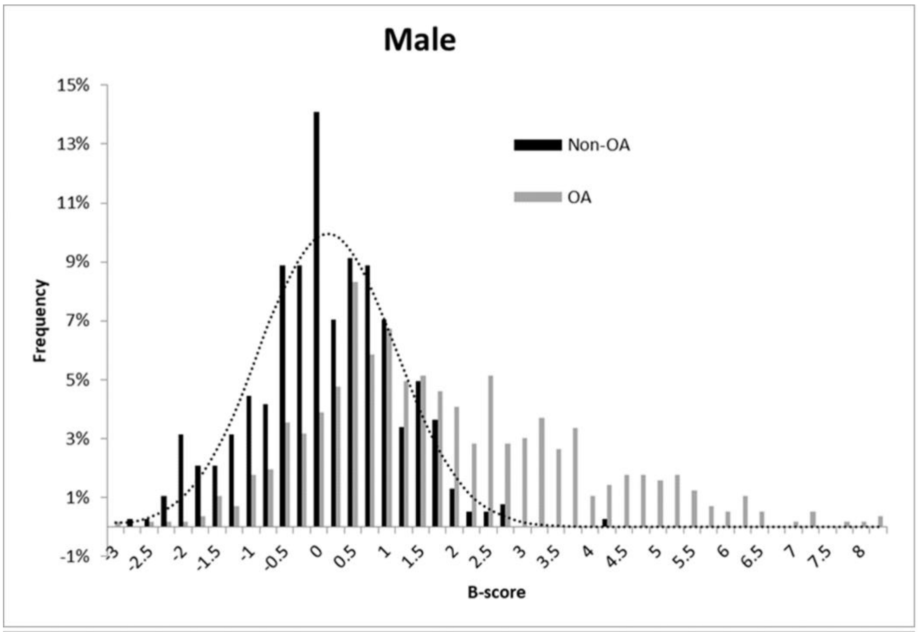

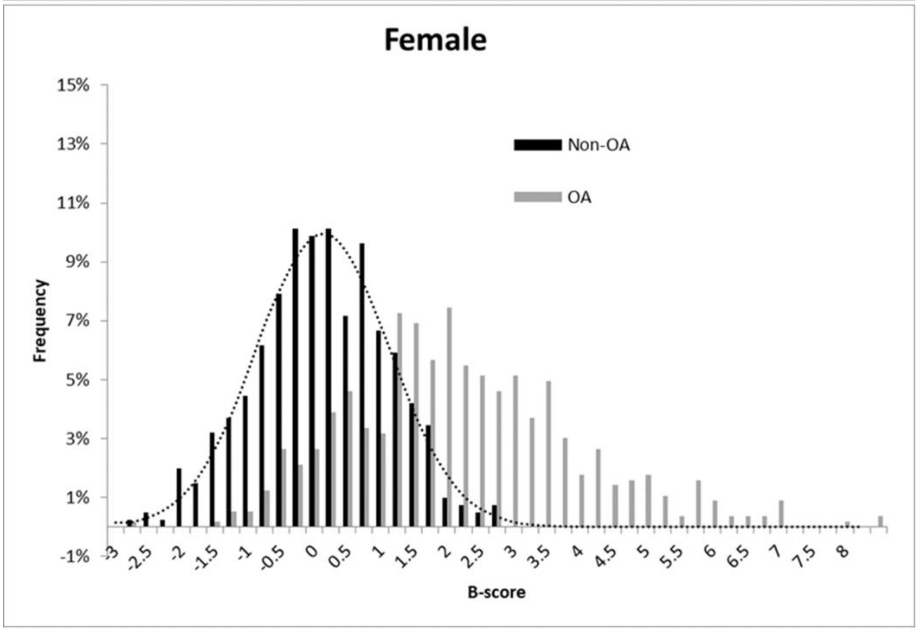

A normal distribution of mean value 0 and a standard deviation of 1 is shown in each histogram using dotted line. Both males and females from the Non-OA group (confirmed KLG0 over 4-year period), are normally distributed along the OA vector, centered on 0 after correction for sex.

REFERENCES FOR SUPPLEMENTARY METHODS

1. Bowes MA, Vincent GR, Wolstenholme CB, Conaghan PG. A novel method for bone area measurement provides new insights into osteoarthritis and its progression. Annals of the rheumatic diseases. 2015 Mar; 74(3):519-525. [Reference number 15 in paper]

2. Williams TG, Holmes AP, Waterton JC, Maciewicz RA, Hutchinson CE, Moots RJ, et al. Anatomically corresponded regional analysis of cartilage in asymptomatic and osteoarthritic knees by statistical shape modelling of the bone. IEEE TransMedImaging. 2010 8/2010; 29(8):1541-1559. [Reference number 16 in paper]

3. Hunter DJ, Bowes MA, Eaton CB, Holmes AP, Mann H, Kwoh CK, et al. Can cartilage loss be detected in knee osteoarthritis (OA) patients with 3-6 months' observation using advanced image analysis of 3T MRI? Osteoarthritis Cartilage. 2010 May; 18(5):677-683. [Reference number 17 in paper]

4. Mahfouz MR, Abdel Fatah EE, Merkl BC, Mitchell JW. Automatic and manual methodology for three-dimensional measurements of distal femoral gender differences and femoral component placement. The journal of knee surgery. 2009 Oct; 22(4):294-304. [Reference not cited in paper]
